# Historical Pedigree Reconstruction from Extant Populations Using PArtitioning of RElatives (PREPARE)

**DOI:** 10.1371/journal.pcbi.1003610

**Published:** 2014-06-19

**Authors:** Doron Shem-Tov, Eran Halperin

**Affiliations:** 1 The Balvatnic School of Computer Science, Tel-Aviv University, Tel-Aviv, Israel; 2 International Computer Science Institute, Berkeley, California, United States of America; 3 Molecular Microbiology and Biotechnology Department, Tel-Aviv University, Tel-Aviv, Israel; Cornell University, United States of America

## Abstract

Recent technological improvements in the field of genetic data extraction give rise to the possibility of reconstructing the historical pedigrees of entire populations from the genotypes of individuals living today. Current methods are still not practical for real data scenarios as they have limited accuracy and assume unrealistic assumptions of monogamy and synchronized generations. In order to address these issues, we develop a new method for pedigree reconstruction, 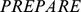, which is based on formulations of the pedigree reconstruction problem as variants of graph coloring. The new formulation allows us to consider features that were overlooked by previous methods, resulting in a reconstruction of up to 5 generations back in time, with an order of magnitude improvement of false-negatives rates over the state of the art, while keeping a lower level of false positive rates. We demonstrate the accuracy of 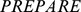 compared to previous approaches using simulation studies over a range of population sizes, including inbred and outbred populations, monogamous and polygamous mating patterns, as well as synchronous and asynchronous mating.

## Author Summary

Learning the correct relationships between individuals from genetic data is a basic theoretical problem in the field of genetics, and has many practical consequences. A wide variety of statistical methods for genetic analysis assume the relationships between individuals are known, and can manifest relatedness information to improve inference. The current state-of-the-art methods for relationship inference consider pair-wise genetic similarity, and use it to infer the relationship between each pair of individuals. Reconstructing the pedigrees of an entire population directly has the potential to use more elaborate relationship information, and thus obtains a better prediction of the familial relationships in the population. In contrast to the full set of pair-wise relationships in a population, genetic pedigrees provide a lossless and conflict-free structure for depicting the relationships between individuals. In an effort to make pedigree reconstruction practical we developed a new method, which is an order of magnitude more accurate than previous methods, and is the first method that has the ability to reconstruct polygamous pedigrees.

This is a *PLOS Computational Biology* Methods article.

## Introduction

Pedigree reconstruction is an important problem in the field of computational genetics, with many potential applications such as genealogy inference, heritability estimation, and victim identification [1]–[4]. Additionally, it has the potential to improve the accuracy of current state-of-the-art relationship inference methods as it uses family structure in a broader sense than just using pairwise genetic similarity information. [5], [6]. There are two main variants of the problem, which require different algorithmic approaches. In the first variant, considered by many classical and contemporary papers, the genotypes of several generations are given, and an attempt is made to estimate the pedigree which best explains the observed individuals, as might be the case in wild animal populations. [7]–[10]. In this paper we consider a more difficult variation of the problem, where we are given the genotypes of the currently living population only, and try to reconstruct the historical pedigree of unobserved ancestors. This variant suits well the scenario of reconstructing the pedigrees of living human populations. [11]. This variant of pedigree reconstruction was previously studied in several theoretical works [12], [13]. These papers focus on presenting theoretical bounds on the length of sequence required for reconstructing pedigrees under various combinatorial and stochastic heritability models, but in contrast to our work, do not aim to provide practical solutions for the problem.

The level of difficulty of the problem is highly dependent on the pedigree in consideration. Particularly, small inbred populations pose a considerable challenge since the probability for multiple mating events within any two families is high, and therefore individual pairs usually have more than two last common ancestors (LCAs). Moreover, in small inbred populations there is a complex relationship pedigree graph due to mating within the family.

Recently, three methods tackling pedigree reconstruction from the genotypes of extant individuals were proposed[11], [14]; these methods assume monogamy, and synchronized generations. Although unrealistic, these assumptions provide a starting point for developing tools that offer useful methodology. The original paper addressing pedigree reconstruction from the genotypes of extant individuals, presented the methods *COP*/*CIP* [11]. 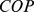 assumes infinite population size, and 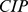 tries to reconstruct the pedigree of small inbred populations. 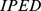 is a follow-up method, similar in principal to 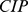, but with improved efficiency [14]. The main idea behind these methods is to construct the pedigree, generation at a time, starting with the given population. In each generation they identify sibling groups using genetic similarity measures, and assign two common parents to each sibling group.

**Figure 1:**
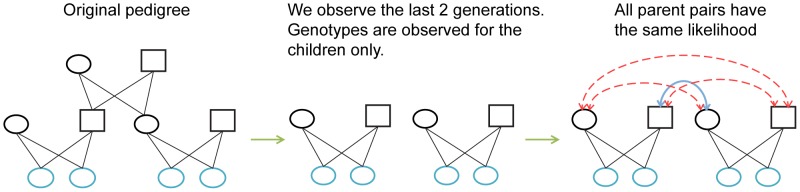
Attempting to reconstruct the simple pedigree on the left, from the genotypes of extant generation (bright blue). Considering observed genetic similarity of extant descendants only, we cannot distinguish which of the four parents in the second generation are siblings (Correctly inferred sibling relationship are colored blue, and wrong potential sibling-relationships in dashed red).

In this work, we point out an important and naturally arising issue of pedigree reconstruction from extant populations, overlooked by all previous methods. We observe that the mother and father of a sibling-group have exactly the same descendants (as must be the case for monogamous couples). Since the genotypes of the parents are unobserved, a pairwise relationship analysis relying on the extant descendants will result in maternal relatives having the same likelihood of being related to the mother and to the father, and vice versa (see Fig. 1). Thus, partitioning the relatives into maternal and paternal relatives is required. Undoubtedly, ignoring this issue has a great potential influence on the quality of inferred pedigrees. We discuss a new framework to help understand and correctly deal with this issue, and present a highly efficient algorithm under this framework - 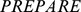 (Pedigree Reconstruction of Extant populations using PArtitioning of RElatives). We extend our method to the case of polygamous pedigrees, and show that our approach results in a considerable improvement in accuracy compared to existing tools, both on monogamous and polygamous pedigrees. Thus, 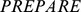 presents a method that is capable of dealing with more realistic pedigree reconstruction problem as compared to previous methods.

## Methods

Similarly to previous methods, we reconstruct the pedigree generation by generation, starting with the last generation, and assuming all of the genotypes of the population come from the same generation. In iteration 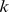, we take the partial 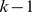 generations pedigree, which we call 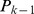, and build 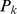 by adding parents to all of the founder individuals in 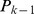. In order to construct the correct pedigree, full-siblings should have two common parents in the pedigree, and half-siblings should have a single common parent. First, we attempt to detect all founder-individual pairs in 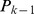 which are most likely to be full-siblings, leaving the detection of half-sibling to a later stage. In previous methods, a sibling graph 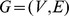 is constructed, where 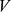 includes the set of all founders in 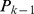, and 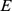 corresponds to the set of pairs of individuals that are likely to be full siblings. Pairs of individuals are considered as potential siblings based on the genetic similarity of the pair's extant descendants. Sibling groups are then detected by finding maximum cliques or proper vertex coloring of the graph 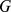. This approach is problematic, since individuals with equivalent descendant sets, such as parent couples, are completely indistinguishable in the graph 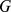 since they have exactly the same set of neighbors. As a result, the siblings graph includes many redundant edges, and fails to represent the true relationship structure.

**Figure 2:**
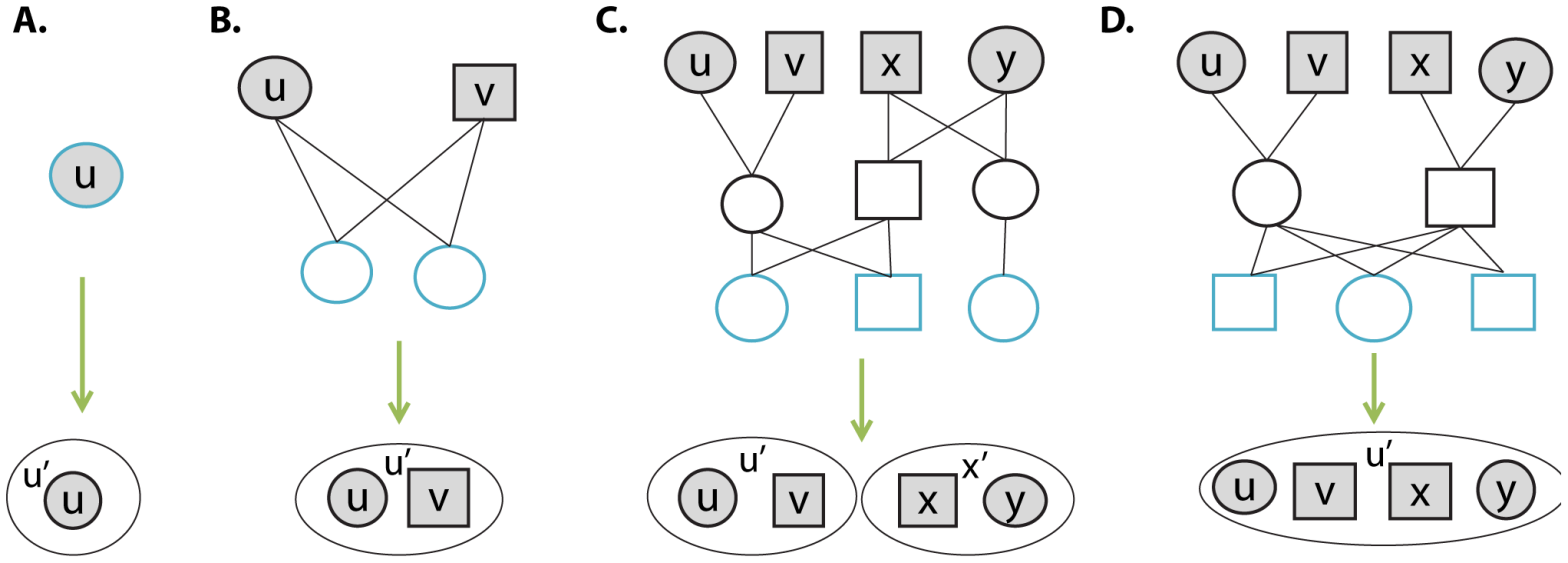
Four examples of vertex contractions, typical for first, second, and third generations. Founders are filled with Grey. Extant individuals are outlined in blue. Green arrows stand for the contraction action.

In contrast with previous methods, we present an alternative graph representation that accounts for the above-mentioned ambiguity, and uses the transitive property of the full-sibling relationship to correctly find the full-sibling groups. We begin each iteration by constructing a contracted siblings graph 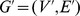. The set of vertices 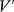 is composed of disjoint subsets of 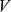. Particularly, each 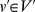 corresponds to a subset of 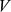, so that for each 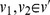 we have 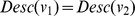, where 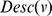 represents the set of extent descendants of 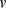 (see Fig. 2). Since vertices of 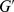 correspond to subsets of 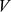, we refer to vertices in 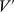 as super-vertices. The set of edges 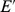 corresponds to potential sibling relationship between the corresponding super-vertices, i.e., 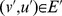 if there are 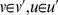 such that 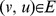. Note that in such case, for every 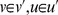, we will have 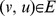. Edges have weights 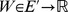 representing the confidence of the relationship. For a vertex 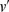, we define 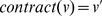 for every 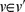. We provide the details for the construction of the set 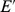 and 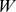 in section 2.1.

The key idea of our method lies in a procedure for the assignment of the edges in 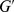 to edges in 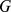 in a consistent way. In principle, we are interested in assigning every super-edge 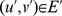 to an edge 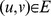 that corresponds to the true sibling pair among all pairs in 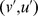. In doing so, we need to take into consideration a set of constraints on the assignments of neighboring super-edges. Ideally, we would like to find the assignment of super-edges to the edges of 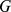, which maximizes the likelihood of the observed population genotypes. In section 2.2, we formulate this problem as an optimization problem using graph terminology, and propose a greedy algorithm which solves it in practice. The assignment algorithm generates an expanded siblings graph 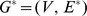, where 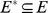, denotes the proposed full-sibling pairs, and forms a disjoint clique-cover of the graph.

Under the monogamy assumption, we finish reconstructing the current generation by adding two common-parents to each sibling clique in 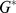. In order to account for potential polygamy we add another step that identifies half-siblings and incorporate these into a second graph formulation. Our approach for the reconstruction of polygamous pedigrees relies on two key observations. First, we note that we can treat the full-sibling relation as an equivalence relation, and the half-sibling relation as a relation between equivalence classes. This is true, since if 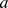 and 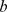 are full siblings, and 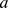 and 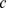 are half-siblings, then 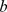 and 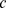 are also half-siblings. According to this observation, we construct a half sibling graph 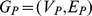 where 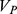 corresponds to the equivalence classes defined by the full-sibling relation, and 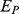 correspond to the half-sibling relation. Second, we observe that the children of every parent in the founder group of 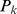 correspond to a clique in 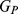. We formulate the half-sibling detection problem, as a second graph optimization problem. To solve it, we develop a heuristic algorithm which attempts to find the maximal-weighted set of edges in 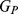. The edge set has to satisfy a set of constraints, which represent natural constraints that govern half-sibling relationships.(see section 2.3).

### 2.1 Constructing the Contracted Sibling Graph

**Figure 3:**
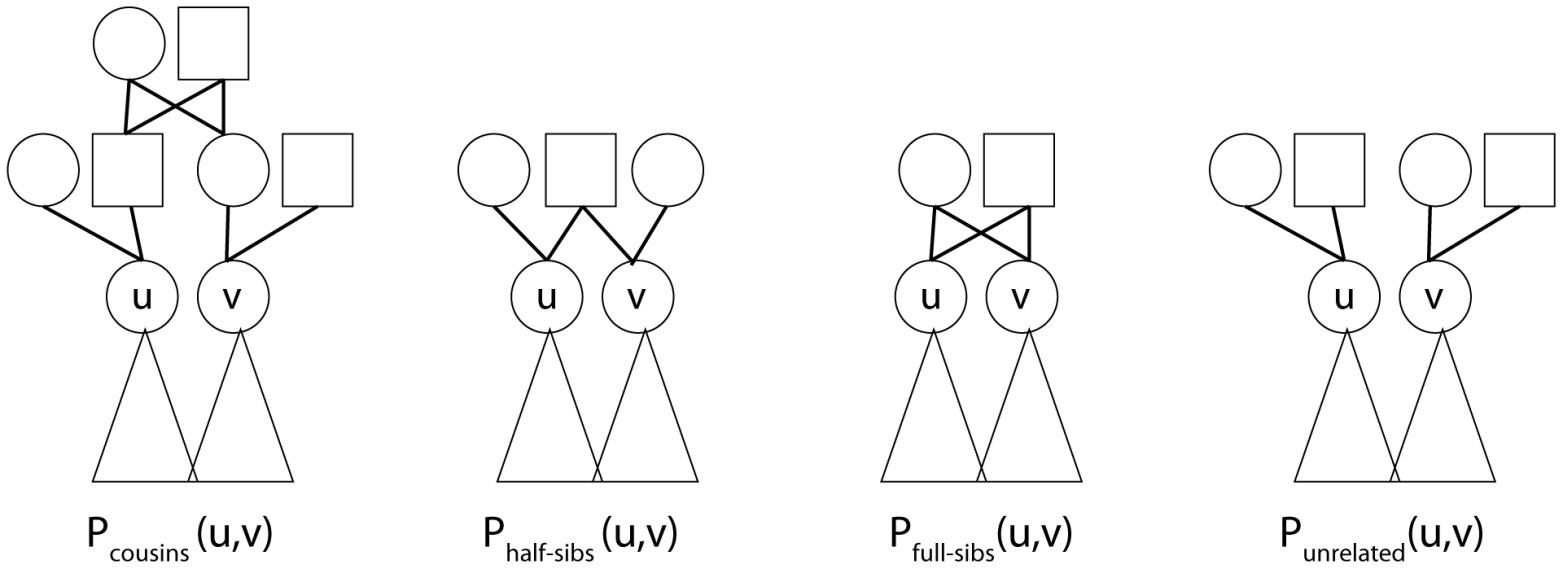
Examples for possible ancestry structures created for individuals 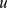 and 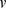 in order to test the relationship between them. The triangles under 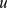 and 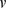 represent their existing descendants, edges represent parent-offspring relationship.

We now describe the construction of the graph 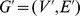. Recall that the set of super-vertices 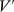 consists of subsets of 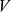 that share the same set of extant descendants. For every pair 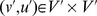 we have to decide whether 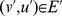. In order to do so, we pick a representative pair 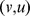, where 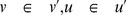, and calculate three scores, corresponding to three putative relations of 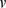 and 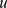: unrelated, siblings, and cousins. For each such relationship 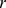, we construct a pedigree 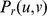 by adding the relevant ancestry structure. For example, when considering the siblings relationship we construct 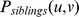 by adding two common parents for 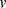 and 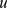. For unrelated pairs we construct 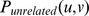 by adding a different pair of parents to each node (see Fig. 3).

We proceed by simulating inheritance on 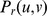; that is, the founders in 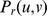 are assigned unique haplotypes and we simulate the recombination process from top to bottom, with a recombination rate of 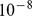. We then calculate IBD segments between each pair of extant descendants in 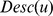 and 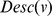 and calculate two *IBD features*: The number of IBD segments, and the total length of IBD sharing (we note that these features of IBD sharing were also considered by 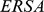 [15], a method for the inference of pair-wise family relationships). We repeat these simulations 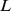 times for a specified parameter 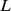, thus obtaining an empirical estimate for the distribution of the IBD features. Using the above empirical distributions, we estimate the probability of observing the IBD features for each pair in 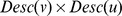 under the relationship 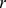. Since the observed IBD features are typically not observed in any of the 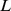 simulations, we use a smoothed form of the distribution using Gaussian kernel smoothing. Formally, let 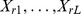 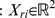 be the simulated IBD features in the 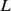 simulations for a hypothesized relationship r. The density 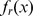 at point 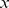 is calculated as:
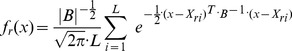



Empirical tests led us to the conclusion that scaling the features to have equal variance and using a diagonal bandwidth matrix 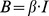 with a parameter 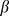 in the range 1 to 8 gives the best results. The parameter 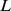 compensates running time and accuracy. The accuracy stops improving near 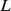 = 50, which ends up with a very efficient analysis (See section 2.4 for more details).

Let 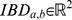 be the observed IBD features between extant individuals 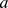 and 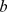. The above procedure results in a probability 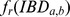, for every 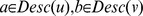 and every relationship 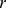 in 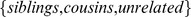.

For each relationship 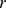, we define
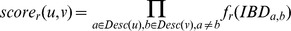



We note that 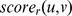 can be intuitively interpreted as a composite likelihood of 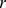. If 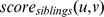 is larger than 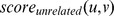 and 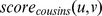 we add 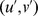 to 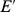 with the weight
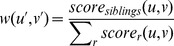



**Figure 4:**
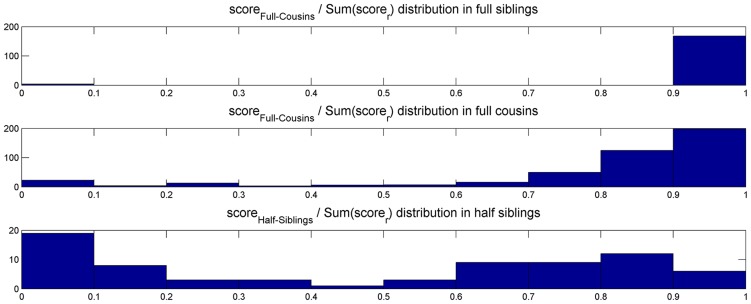
Distribution of relationship scores under specific true relationships.

Fig. 4 shows the distribution of 
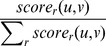
 under different true relationships. Notice that cases where 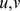 are distantly related (cousins, 2nd-cousins etc.) will tend to have a maximal score under 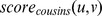. This is desirable, since we only seek to distinguish siblings from non-siblings at this point.

### 2.2 The Assignment Algorithm

In the assignment stage, we are given the contracted siblings graph 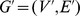, and we search for an assignment of a sibling relation between super-vertices, depicted by an edge 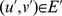 to a single sibling-relation between two individuals 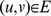. Our assignment needs to obey the transitivity constraint of the full sibling relation. Recall that the weight of an edge 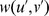 corresponds to the strength of evidence for the existence of a sibling pair 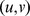, where 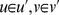. We therefore formulate the edge assignment problem as follows:

#### *Problem 1.* Maximum weight disjoint clique cover edge assignment

Given the contracted graph 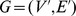, find the maximal-weight set of edges 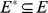, such that 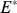 is a legal assignment of 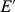, under the constraint that the set of assigned edges 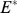 forms a clique cover of the graph 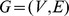, i.e., 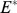 is composed of an edge-disjoint set of cliques.

We first show that the above problem is NP-hard:

#### Theorem 1

*The maximum weight disjoint clique cover edge assignment is NP-hard.*

*Proof.* We will show a reduction from maximum clique. In [16] it is shown that it is NP-hard to decide whether a graph 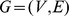 has a clique of size 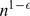 or if its largest clique is smaller than 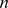, where 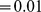. Consider an instance 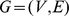 to the clique problem, and let 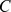 be its largest clique. We define 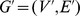, where 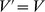, and 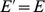. Thus, any clique cover of 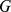 is a legal assignment of 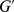. Note that if 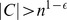 then the optimal clique cover is necessarily of size at least 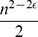. On the other hand, if 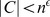 then it is easy to see that the optimal clique cover is obtained in case all cliques in the cover are of size 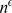, and thus the clique cover size is of size at most 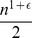. Thus, if the Maximum Weight Disjoint Clique Cover Edge Assignment was polynomial, then we could decide in polynomial time between the case where the maximum clique is of size 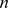 and the case where the maximum clique is of size 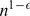, which is an NP-hard problem.

We therefore apply the following greedy algorithm. We will need to introduce a few notations. First, we treat vertices 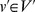 as vertices in 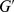, as well as subsets of 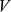, depending on the context. For each 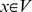, we denote by 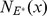 the set of neighbors of 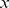 in 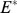. Moreover, we define 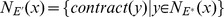, i.e., the set of super-vertices corresponding to the neighbors of 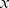 in 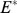. Finally, let 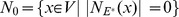.

We start by setting 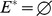. The algorithm proceeds by traversing all super-edges 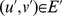 in decreasing weight order. In each iteration the set 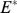 consists of a set of disjoint cliques of 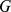, and 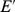 consists of a set of yet to be assigned edges. For each 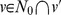 and 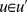 we say that 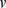 can be added to the clique of 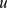 if for every 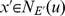 we have that 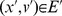. Similarly, we say that 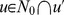 can be added to the clique of 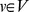 if for every 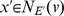 we have 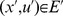. When traversing an edge 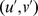 we search for a pair 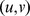 where 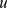 has the maximal clique size, 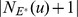, from within 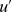, 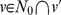, and 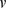 can be added to the clique of 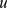 (or in a symmetric manner that 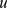 can be added to the clique of 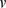 and 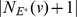 is maximized). We then assign 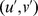 to 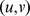 by adding 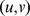 to 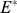, and removing 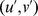 from 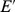. We also assign 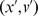 to 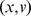 for every 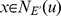.

**Figure 5:**
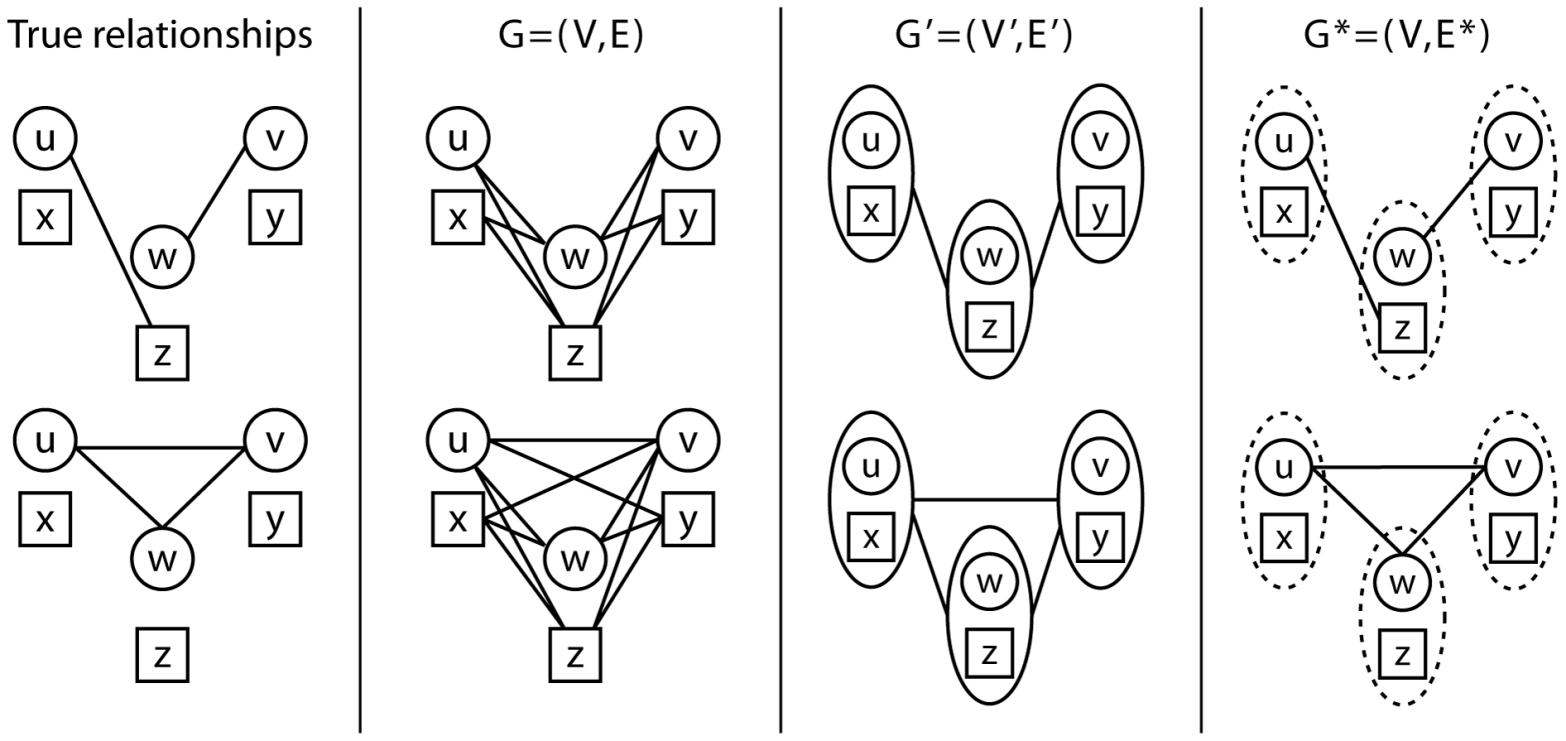
Intuition for sibling assignment, depicting the potential-siblings graph 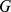, the contracted graph 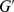, and assigned graph 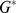. In both examples 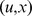,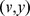,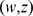 are parent couples with extant descendants in the observed population. A. For the case where 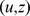,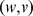 are full-siblings, the contraction will end in 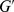 composed of three super-vertices, connected by two edges; the assignment algorithm will assign each edge to a disjoint clique. B. If 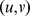 are also full-siblings, a 3-clique is formed in 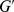; the assignment algorithm assigns all edges to a corresponding 3-clique of siblings.

Fig. 5 summarizes the contraction and assignment stages with an example. Note that cases such as 3-cliques in 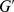 (Fig. 5-B) can have multiple assignments with the same score (3 siblings from one parent couple, or 3 pairs of siblings from 3 different parent couples). In such cases our algorithm chooses the more parsimonious solution in which there is a smaller number of parents.

### 2.3 Half-sibling Detection

In the following stage we define the half-sibling detection problem, where we attempt to detect groups of individuals with a single common-parent. First, we define the full-sibling relation, on individuals: 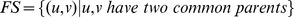. Notice that 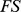 is defined as being reflective, and thus it is an equivalence relation on 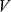. 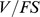 is the quotient set of 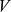 on 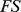, which in this case is simply the set of disjoint groups of full-siblings. We obtain 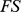 from the edges in 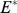 computed in section 2.2. 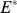 is a clique cover, and so naturally describes an equivalence relation.

**Figure 6:**
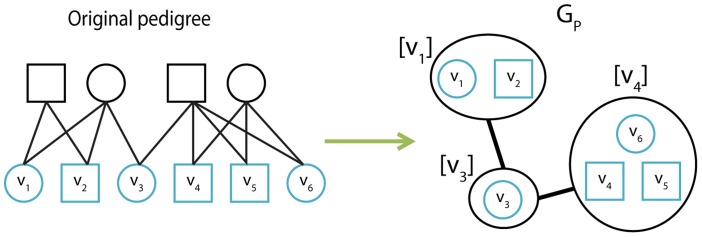
An example for the construction of 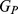 in the first generation.

We define 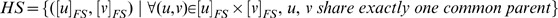, which is the half-sibling relation, as a relation between equivalence classes in V, in respect to 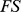. Assuming the pedigree is known, HS is defined properly since if 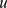 and 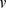 are full siblings, and 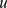 and 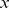 are half-siblings, than 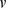 and 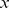 are half siblings. This allows us to simplify the half-sib detection problem, by constructing the polygamy graph 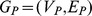, where 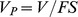 s.t each vertex 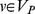, represents a group of full-siblings, and each edge 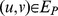 represents a half-sibling relation between 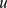 and 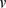 (see Fig. 6). The edges are added to 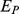, with a similar stage to 2.1, only the hypotheses tested this time are made for siblings groups 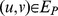, and are relevant to the half-sibling case (half-siblings,cousins,unrelated).

The graph 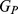 has the convenient property that if a group of individuals 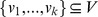 have a single-common-parent then 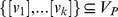 form a clique in 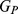. We thus assume by parsimony, that each clique 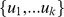 in 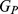 connects all of the children of a single parent 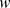, such that each 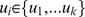 is a full-sibling-group which contains the children of 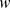 and a single mate. We therefore formulate the half-sib detection problem, as follows:

#### *Problem 2.* Maximum weight, two-color clique cover

Given the graph 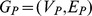, find sets of edges 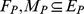, such that both 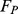 and 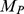 consist of an edge-disjoint set of cliques, 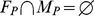, and the total weight of 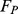 and 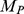 is maximized.

#### Theorem 2

*The Maximum Weight Two Color Clique Cover is NP-hard.*

*Proof.* We will show a reduction from maximum clique. Consider an instance 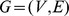 to the clique problem, and let 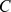 be its largest clique. If 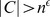 we can set 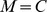 and 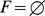, and therefore the optimal solution to the coloring problem has at least 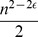 edges. On the other hand, if 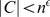 then the size of each of 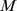 and 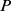 is at most 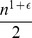, and thus the total size of both of them is bounded by 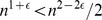. Thus, by solving the Maximum Weight Two Color Clique Cover in polynomial time we can decide between graphs with clique size at most 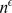 and graphs with clique size at least 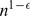, hence the problem is NP-hard.

Informally, we try to color all edges 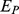 in two colors, 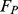 and 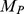, s.t each color creates a set of disjoint cliques. 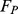 colored cliques, represent full-sibling-group cliques with a single common father, and 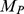 colored cliques, represent full-sibling-group cliques with a single common mother.

This problem is also NP-hard and we therefore use the following greedy approach. For simplicity, we assume 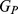 is connected. The algorithm begins by setting 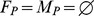. We will denote by 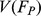 and 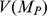 the set of vertices induced by 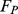 and 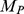 respectively. The algorithm proceeds in iterations. In each iteration we search for the heaviest clique 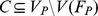 such that 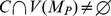, and the heaviest clique 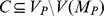 such that 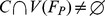. Without loss of generality, assume that the heaviest among those is a clique 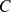 in 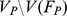. If 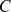 contains only one vertex, we search instead for the heaviest clique 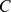 in 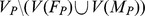. We add the edges of 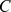 to 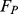 and remove these edges from the graph. Clearly, both 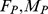 consist of a set of disjoint cliques of 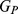.

**Figure 7:**
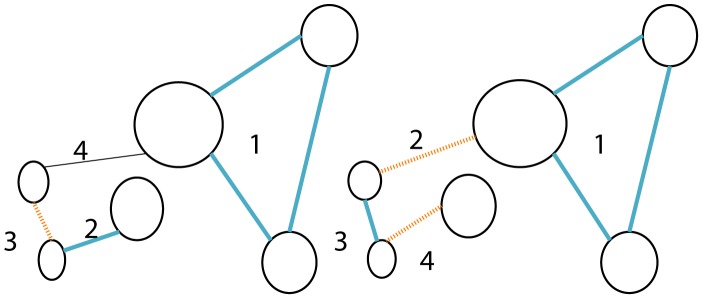
An example for a case where the coloring order we purpose enables coloring more cliques with two colors than coloring the same graph with an arbitrary order. The coloring order is depicted near the cliques. In the left graph we follow the depicted order and color the clique blue if possible, else we color it dashed-orange. The fourth click cannot be colored since it touches a blue and a dashed-orange clique. In the right graph we use our coloring scheme, which prefers coloring cliques touching cliques that are already colored. Using this order we are able to color all four cliques.

Notice that we try to minimize the number of arbitrarily colored cliques, by choosing cliques adjacent to cliques that are already colored. Simulation studies show that choosing this coloring order increases the half-sibling sensitivity from 85% to 97% on average (see table 1). It is easy to see that sub-graphs that are composed of a connected list of cliques will be colored optimally by our coloring scheme. An example for such a graph is depicted in Fig. 7.

**Table 1:** Sensitivity and PPV scores (as defined in the results section) of half-siblings using two coloring order schemes.

	PREPARE		naive	
Population size	Sensitivity	PPV	Sensitivity	PPV
200	1.0	0.91	0.91	0.91
300	0.91	0.88	0.79	0.85
400	0.97	0.88	0.85	0.88
500	1.0	0.88	0.88	0.91

(1) PREPARE's greedy coloring scheme as described in section 2.3. (2) Coloring cliques from the heaviest to lightest; if possible color with 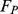, else if possible color with 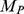.

The graph formulation of the half-sibling detection assumes that each edge in 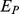 represents a unique half-sibling relationships. We notice, that in some cases 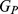 might contain redundant edges. In order to simplify the explanation, we extend the definition of 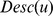 to nodes in 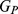: 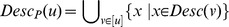. The problem arises, when there exists a pair of nodes 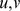 from the same generation, such that 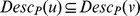. In such a case, an edge 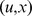 may be added to 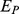, as a result of a relationship 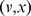. Trying to contract 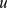 and 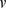 is not sound, since different relationships can be detected for 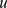, and 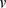 to a third vertex 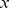, by testing them separately. Instead, we apply a preprocessing to 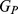, in the form of a set of parsimonious rules. The rules aim at filtering all the edges, except the ones that explain the observed features in the simplest way.

**Figure 8:**
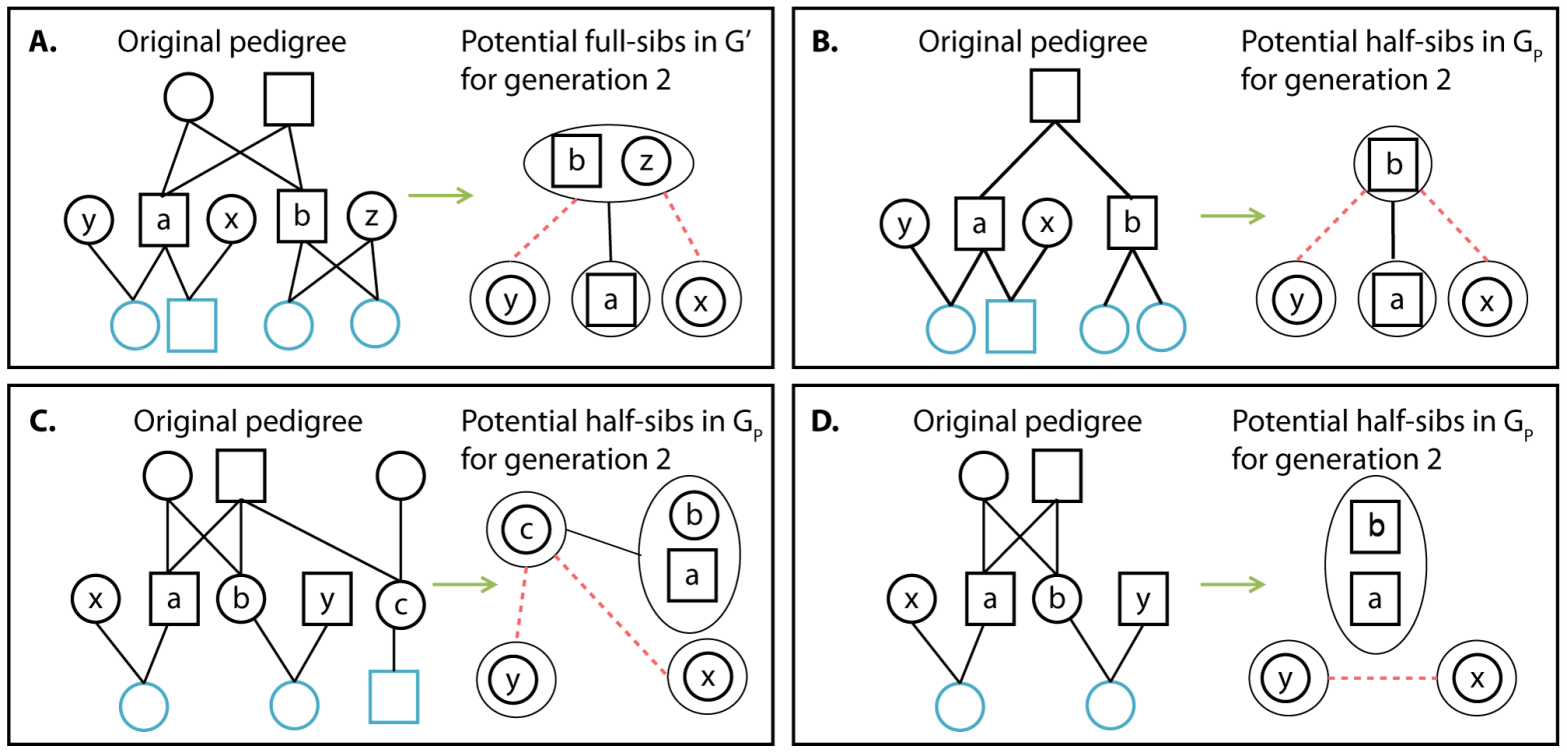
Depicting cases where edge removal rules are required in polygamous pedigree reconstruction. Redundant graph edges are dashed red, correct edges in solid black.

The first rule we apply concerns the case depicted in Fig. 8-A. In this case, an individual 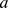, with a half-sibling 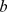, has children with two mates 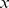, and 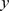. Since 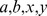 do not have full siblings, each of them is represented in 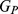 as a sibling-group of one individual. Since 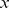 and 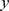 have children only with a, their descendant sets are contained in 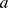
's descendant set. As a result, half-sibling edges should form between 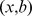 and 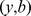, additionally to the correct edge 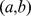. To deal with this case, if we find a node a, in 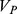 that has two mates, 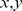 and the following holds: 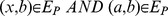, we remove 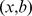 (we do the same for 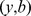). A similar rule is applied to the contracted graph 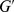, where redundant full-sibling edges result from an equivalent case to the one just mentioned, and are removed in the same manner (see Fig. 8-B). A third rule is applied to 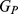 to deal with a case similar to the one in rule 1, only the mates 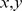 are not the mates of a single individual 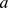, but instead 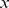 is the mate of 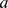, 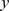 is the mate of 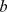 and 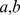 are full-siblings (see Fig. 8-C). In such a case, a true relation 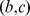 may cause redundant half-sibling edges 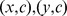. These cases are characterized by mates 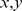 that have few or no full-siblings. Thus, we look for edges 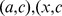) where 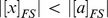, such that 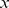 is the mate of 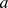, and remove 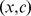 from 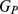. Finally, we observed half-sibling edges forming between two mates 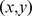, of 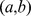 such that 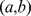 are full-siblings. This results from the fact that most of 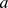 and 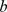
's descendant similarity was already explained by the formation of the full-sibling relationship 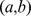. The difference between the half-sibling hypothesis and the null hypothesis for 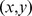 becomes small. As a result, noisy decisions are made. To handle this final case, we remove half-sibling edges between mates of full siblings 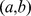 if they have a half-sibling edge 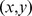 in 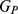
(see Fig. 8-D).

### 2.4 Efficiency Considerations

Simulating inheritance for the descendants of every two individuals during the graph constructions is very time consuming, and is the reason 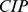 is impractical for large populations, or pedigrees deeper than 4 generations. Notice that if a pair of extant descendants has exactly the same ancestor structure in the pedigree, than the simulated IBD features are sampled from the same distribution. 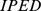 purposes caching individual pairs with identical inheritance paths, and introduces an accompanying dynamic programming algorithm for minimizing the number of operations.

In 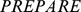, we use a simplified version of this idea. For every pair 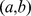 of extant descendants, we calculate a least-common-ancestors (LCAs) vector 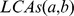, which is a list of the meiosis distances between 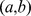 and their least common ancestors. For example, all full-siblings will have the 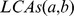 = [1,1], since full-siblings always have two common ancestors, with one separating meiosis. We hash the simulated distribution for this LCA vector, where the key represents the vector itself, and the value is the distribution. We simulate inheritance only when needed, i.e. when 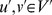 have at least one descendant pair, without a hashed distribution, thus saving most of the redundant computation. Practically, the running time of 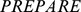 is equivalent to the running time of 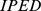, and is even slightly faster (see Table. 2). Although 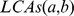 does not capture completely the ancestry structure for 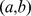, we observed empirically (data not shown) that running simulations for each ancestry structure does not improve the reconstruction accuracy. Apparently, pairs of individuals 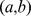 with the same LCAs vector have similar IBD distributions. The similarity is large enough to make the repetition of inheritance simulation for two such pairs redundant.

**Table 2:** Running times of PREPARE on 1.6GHz Intel Core i5-2467M machine with 4G RAM using a single thread.

Population Size	monogamous	polygamous
100	31s	4m 18s
200	53s	9m 21s
500	4m 55s	56m 40s
1000	10m 27s	93m 41s

The two parameters affecting the running time of prepare is the population size, and whether PREPARE is run on monogamous or polygamous mode. Most of the running time is spent on reconstructing the fifth generation.

### 2.5 Availability

The 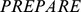 method, inheritance simulators, and quality evaluation tools are available at http://www.cs.tau.ac.il/ 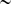
heran/cozygene/software.shtml

## Results

We compare the accuracy of our method to previous pedigree reconstruction methods on numerous simulations. Different simulations include combinations of population size and inheritance modes (monogamous and polygamous). Smaller population sizes correspond to inbred populations with multiple relationships between families. Larger populations correspond to outbred populations, with simpler pedigree structures. We also study the effect of population bottlenecks on the reconstruction quality. In order to test 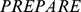 on a more realistic scenario, we run it on a realistic simulation starting from HapMap phaseIII 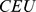 and 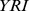 populations as founders. The simulation simulates polygamous random mating in this population for 200 years, reaching to a final population size of 1000. Finally, we apply PREPARE on the HapMap 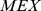 population as a feasibility test for application of our method for real populations.

### 3.1 Simulations

Similarly to previous methods, we use a Wright-Fisher (WF) simulator that includes recombination and genders. We add several new features, which makes this simulator more flexible. First, we add the ability to control polygamy through a polygamy probability parameter 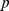, which controls the probability for an individual to have a child with more than one mate. Second, we add an option to simulate dynamic population sizes by specifying an initial population size and a final population size. The simulator calculates the required population change per generation and modifies the population size with that ratio in every generation.

Additionally, we experiment with a more realistic forward simulator that does not assume synchronized generations, and allows polygamy. We simulate inheritance as a function of time, where individuals can have children after the age of 20, and die at an age drawn from a capped exponential distribution with mean 50. The birthrate is changed according to the current population size, and is tuned to reach a predefined target population size. This simulator produces actual recombined haplotypes, from the haplotypes of 160 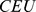 and 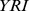 HapMap representatives. More specifically, the simulation runs in 5 year iterations, and a pool of unmated mature individuals is maintained at all times. Every iteration, individuals from the pool are matched to uniformly drawn mates. A matching has probability 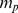 to succeed. Every mated pair has a probability 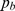 to have a child, where 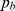 is initialized to be 1, and is modified in every iteration by +0.2 or -0.2 depending on whether the current population size is smaller or larger than the target population size. Polygamy is achieved through second-marriage, which can occur since once a mate dies, the individual is added back to the unmated pool. Finally, in order to include possible IBD detection errors, we detect IBD segments from simulated genotypes using 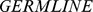, [17], and extract the IBD-features information from its output. This simulator also has the advantage of having a possible dynamic population size. The population grows or shrinks depending on the initial and target population sizes.

### 3.2 Quality Evaluation

**Figure 9:**
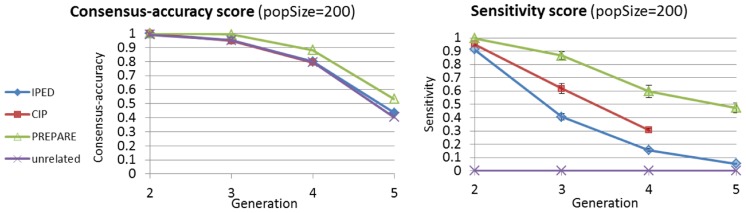
Example for the problematic nature of the consensus-accuracy score, in contrast with the sensitivity score we propose. Notice how the unrelated pedigree structure receives similar consensus-accuracy scores to 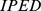 and 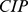 reconstructions. Still, 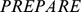 scores are significantly higher. Shown are average scores over 5 simulations, and standard deviation bars. (Some error bars are too small to be visible).

Many different measures can be accounted in evaluating the quality of reconstructed pedigrees. We first use a previously defined score, to compare 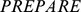 to previous methods. For the large part of the presentation, we define and use other natural evaluation scores, which we deem as more relevant, and interpretable. In previous methods, a consensus-accuracy score, which counts the number of extant individual-pairs with the same minimal meiosis-distance as in the true pedigree was used [14]. This score treats correct detection of unrelated pairs and related pairs identically. This is problematic since the number of unrelated pairs dominates the score. For example, a trivial algorithm that outputs a pedigree where all individuals are unrelated receives a high consensus-accuracy score (see Fig. 9). As a new standard for pedigree-reconstruction evaluation, we suggest three types of scores: sensitivity, positive-predictive-value (PPV), and IBD-length prediction error.

We define sensitivity as the fraction of correctly constructed (distance wise) related pairs from the total number of related pairs in the original pedigree. PPV is defined as the fraction of correctly constructed related pairs from the total number of related pairs in the reconstructed pedigree. More formally, define 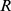 as the reconstructed pedigree, 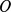 as the original pedigree, 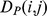 as the minimal number of meiosis separating 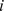 and 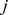 in pedigree 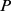, and 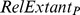 as the set of extant-individuals, which are related according to pedigree 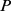. Let 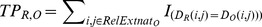. Then,
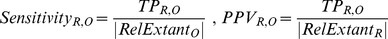



We run 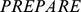 for 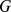 generation, and compare the scores of reconstructed pedigrees for every generation 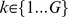 against the first 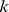 generations of the original pedigree. This way we can assess the accuracy of different relatedness degrees (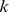 = 2 corresponds to siblings, 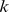 = 3 to siblings and first-cousins, etc.)

Scores such as sensitivity and PPV have the disadvantage of not weighing mistakes according to their magnitude. A second disadvantage is that the minimal meiotic distance does not capture the full complexity of a real pedigree (for example, double cousins detected as cousins will get a full scoring). For these reasons, we suggest to alternatively measure pedigree quality by calculating the root mean square IBD-length error (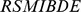):
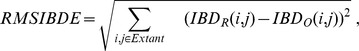
where 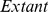 is the set of extant individuals in the population, 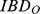 is the observed total length of IBD segments between individuals 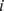 and 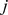, and 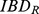 is the total length of IBD segments between individuals 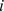 and 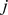, as given from simulating inheritance on the reconstructed pedigree 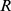. Since this score is dependent on the randomized scoring-simulation, we average the score of 5 runs. The 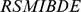 can be interpreted as the expected prediction error (in Mbp) of the typical pair-wise total-IBD-length, given the reconstructed pedigree.

### 3.3 Comparing 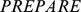 and Competing Methods on Monogamous Simulations

**Figure 10:**
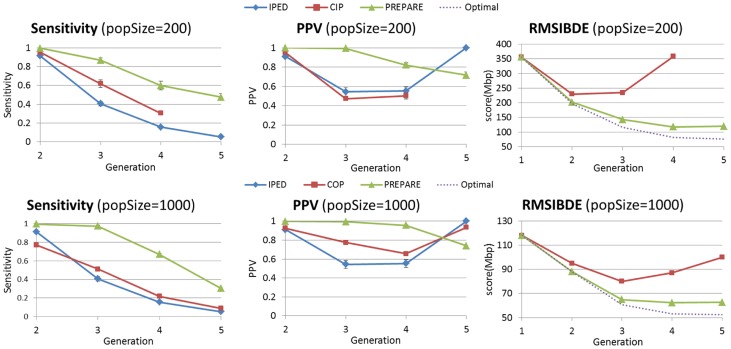
Comparison of pedigree reconstruction methods for monogamous populations, using Sensitivity, PPV, and 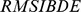. Populations were simulated with Wright-Fisher simulations of 5 generation. Shown are average scores over 5 simulation, with standard deviations bars. The optimal 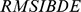 score is calculated by scoring the true k-generation pedigree. The first generation pedigree in the 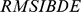 figures, is the score of the pedigree where all individuals are unrelated, and is shown as reference. (Some error bars are too small to be visible).

We tested the competing methods on monogamous Wright-Fisher simulated population, of constant sizes: 100, 200, 500, and 1000. When it was possible, we ran 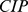 (up to 4 generations due to its high runtime complexity), and for larger populations we ran 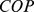. 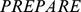 was run in monogamous mode. Results on 100 and 200 individuals were similar, as well as results for 500 and 1000 individuals. In Fig. 10, we compare the three methods for small populations (200) and larger populations (1000). In all the scenarios we tested, 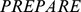 was the most sensitive; for pedigrees of up to 5 generations (corresponding to 3rd cousins) and populations as small as 100 individuals. For the larger populations, the improvement in sensitivity is highest, where 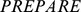 is able to build a pedigree which correctly predicts the minimal meiosis distance of more than 95% of 1st and 2nd degree relatives and more than 60% of relatives up to 3rd degree. At the same time, 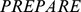 has a higher PPV up to pedigrees of 4 generations. In the 5th generation it gets a lower PPV than the other methods, but this disadvantage is not meaningful, since the sensitivity of these methods in the 5th generation is very low. 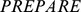 gives better quality of results for larger populations, which is natural, since they tend to form simpler pedigrees with less multi-relationships between families, and less inbred families.

Considering 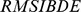 scores, 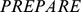 gets much better scores than the second best method, and is close to the optimal score, especially for larger populations. 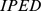 gets worse 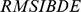 scores than 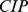/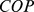 as a result of its practical tendency to over-predict inbreeding, which we observed during our experiments. An important feature of 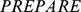
's score is that it is non-increasing in the number of generations, similarly to the optimal score. In contrast, we do not see this behavior in other methods. Interestingly, the optimal scores decrease as the population size increases. We attribute this mainly to the increasing proportion of unrelated pairs in larger populations, which are easier to predict.

### 3.4 The Effect of Population Expansion on the Success of Pedigree Reconstruction

The simplified Wright-Fisher model that was used in pedigree reconstruction methods up to this day assumes a constant population size. Real populations sizes are obviously not constant, and it is known that population bottlenecks and expansion affect the IBD distribution in the population. We have conducted an experiment to test the effect of population size shifts on the distribution of chosen IBD features, and as a consequence on the quality of the resulting pedigree. We have run the Wright-Fisher simulation with changing initial population sizes of 100,200,300,400,500 and fixed the final population size at 500. By looking at the distribution of IBD features between all pairs of individuals, it is clear to see that the number of IBD segments and the mean IBD segment length have an inverse relationship with the initial population size. This corresponds to a higher proportion of relatives in the populations with smaller initial size. We have found that populations that grow from 100 to 500 individuals in five generations have similar IBD feature distributions to populations with constant population size of size 200. Interestingly the quality of the resulting pedigree of these populations remains unchanged when the initial population size is gradually decreased from 500 to 200. Only at initial size of 100 does the quality decrease. Sensitivity levels for initial population size of 100 are 0.96,0.75, and 0.54 for 2,3 and 4 generations. The largest decrease is for 3-generation pedigrees where the sensitivity is decreased by 10% on average. The PPV remains above 0.95 for generation 2,3 but is decreased from 0.85 to 0.71 in generation 4.

### 3.5 Comparing 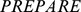 and Competing Methods on Polygamous Simulations

**Figure 11:**
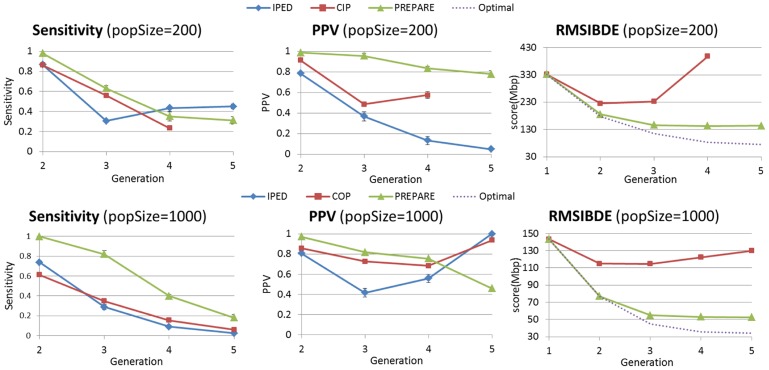
Comparison of pedigree reconstruction methods for polygamous populations. Populations were simulated with polygamous Wright-Fisher simulations of 5 generation. Shown are average scores over 5 simulation, with standard deviations bars. (Some error bars are too small to be visible).

To asses the quality of 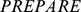 on polygamous populations, we simulated polygamous populations of sizes 200 and 1000 with the Wright-Fisher model. In the simulated populations 33% of the siblings are half-siblings on average. Details regarding the execution of previous methods are the same as in section 3.3. 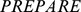 was run with the polygamous mode. The results are summarized in Fig. 11. Once again 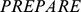 is generally superior in terms of sensitivity, PPV and 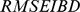. A notable exception is 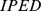
's relatively high sensitivity in generations 4 and 5 in smaller population sizes (200). Note however that this sensitivity comes at the cost of very low PPV and very high 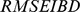 in these generations. The 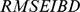 of 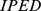 is not shown in the graph since it is out of the charts, getting as high as 1500 Mbp. This result suggests that 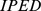 has a strong tendency to over-predict relationships in small polygamous populations.

**Figure 12:**
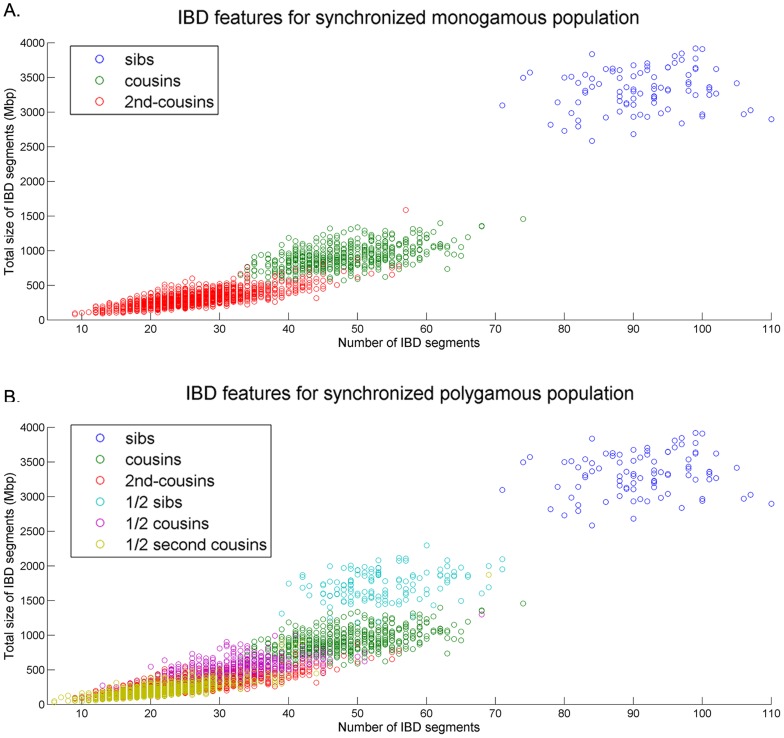
Simulated IBD feature distribution in monogamous and polygamous populations. The overlap in polygamous distributions is the main challenge in reconstructing pedigrees of real populations.

Similarly to the monogamous case, 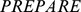 achieves higher performance on larger, and as a result, more simply related populations. For a population size of 1000, 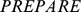 is able to build a polygamous pedigree which correctly predicts the minimal meiosis distance of more than 97% of 1st degree relatives and more than 80% of 2nd degree relatives while maintaining a PPV greater than 80%. Polygamous populations pose a much greater challenge for pedigree reconstruction, and the performance is decreased in comparison to monogamous populations. According to our analysis, the difficulty in reconstructing polygamous pedigrees stems from the fact that the IBD feature distributions for the range of possible polygamous relationships have greater overlap than in monogamous relationships (See Fig. 12).

### 3.6 Reconstructing Realistically Simulated HapMap Descending Population

**Figure 13:**
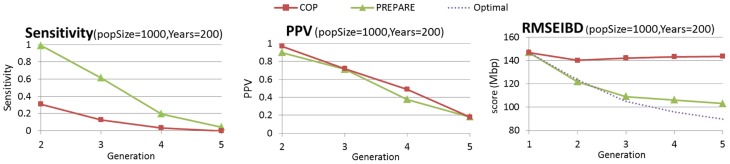
The performance of PREPARE on realistic simulation is comparable to polygamous Wright-Fisher simulations. The simulated population grew from 160 individuals of the 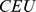 and 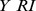 HapMap populations to 846 individuals in 200 years. This simulation accounts for IBD detection errors, asynchronous mating and dynamic population size.

We test the performance of 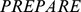 on populations produced by the polygamous, asynchronous forward simulator. We run the simulator for hundreds of simulation years, resulting in the mixing of the different generations, and reconstruct the last five generations. We use un-phased IBD segments, to account for the fact that our input is genotypes, and not haplotypes. As a necessary step, we aim to filter out cross-generation relationships, which are not currently modeled, by taking the genotypes from the youngest age stratum (Ages 0-20). We used the 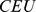 and 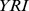 HapMap genotypes as the founder population for our simulation. The results show a comparable success to the Wright-Fisher simulation, increasing our confidence that 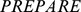 can be run on real populations. All accuracy measures show a decrease in accuracy compared to the Wright-Fisher simulation results. This is expected due to the addition of several factors (as discussed above), which adds to the complexity of the analysis (see Fig. 13).

### 3.7 Application for the HapMap MEX Population

We next use 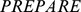 to reconstruct the historical pedigree for the HapMap MEX population. This population is of interest to us since it is known to contain several relatives, including a single 4-generation pedigree [5]. Age information is not publicly available for this dataset. Instead, we use known parent-offspring relationships to separate the population into three generations. The correct pedigree is not known, so we use previous relationship inference results by Stevens et al. to validate our results[18].

**Figure 14:**
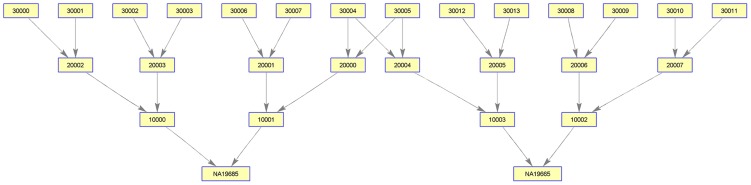
PREPARE successfully isolates the 4 generation pedigree found by CARROT. Nodes correspond to individuals, and edges to parent offspring relationships. The last generation individuals are real HapMap individuals, and the other nodes are ancestors predicted by PREPARE.

Running 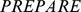 on the parent generation of HapMap phaseII+III 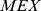 genotypes, we are able to detect a single sibling relationship (NA19662,NA19685), three first-cousin relationships (NA19662,NA19664), (NA19664,NA19685), (NA19657,NA19786) and two second-cousin relationships (NA19657,NA19785), (NA19785,NA19786). We are able to reconstruct correctly the pedigree found by Kyriazopoulou et al. We do this fully automatically and without using the genotypes of the two known grandparents: (NA19662,NA19685) which makes the reconstruction a significantly harder task(see Fig. 14). Further more, all of the relationships inferred by 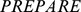 except (NA19785,NA19786) are confirmed by Stevens et al.[18]. (NA19657,NA19786) are inferred as Third degree instead of first cousins, and (NA19657,NA19785) as Unknown degree instead of second cousins.

## Discussion

In this paper, we take a step towards making pedigree reconstruction from present living populations, a realistic objective. By developing better quality assessment tools, we were able to come to the conclusion that our method reconstructs pedigrees with significantly higher quality then previous methods, and in comparable running times. 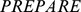 is the first method to our knowledge to address polygamy, and paternal/maternal relative partitioning. Although we succeed partitioning the relatives, there is no way to know which relatives are really related to the father, and which to the mother by considering autosomal data alone. We are not worried about this lack in specificity, as we do not strive to learn the ancestral genders. Instead, we are interested in inferring the pedigree structure, which provides the relatedness structure. Our graph framework, brings to the surface several ambiguous cases that cannot be solved without utilizing additional subtle information. For example, the assignment of a 3-clique (see Fig. 5-B) might be decided better by considering three-way IBD sharing. The chance of having triple IBD sharing diminishes much faster than the chance of pair-wise IBD sharing and limits the theoretical possibility to correctly reconstruct these cases in advanced generations. Reconstructing inbred relationships correctly remains an unmet challenge by all methods in the present. It seems that an approach to deal with inbreeding will need to utilize additional inbreeding imprints on the data, such as homozygosity levels and other IBD-features not used today. Additionally, current methods do not include inbreeding options in the hypothesis testing stage, which might lead to the wrong conclusions when inbreeding exists. Despite the above, our method is able to reconstruct high quality pedigrees by dealing correctly with the most frequently arising cases in randomly mating populations. We believe that improving the performance on such rare aspects will probably have a small impact on the pedigree quality. More importantly, in order to further improve the reconstruction quality of polygamous populations, it seems that a better set of IBD features needs to be found, with higher separating power between different relationship types. Theoretically, the size of a family can influence the scores of its founders since larger families will contribute more extant individuals to the score computation. Simulating populations with differing typical family sizes show little effect on the quality of reconstruction. The current 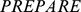 method can be applicable for real populations, with the setback that only a specific age-range must be taken as input, such that most inter-generation relationships will be excluded.
